# In Vivo Emergence of Oxacillin Resistance in mecA‐Negative MSSA Endocarditis With Metastatic Infection

**DOI:** 10.1002/ccr3.71888

**Published:** 2026-01-19

**Authors:** Leandro Bosch, Alexie Carletti, Toluwalase Awoyemi, Mohamed Al‐Kazaz, Alexander J. Nemeth, David Young

**Affiliations:** ^1^ Northwestern University Feinberg School of Medicine Chicago Illinois USA; ^2^ Division of Hospital Medicine, Department of Medicine Northwestern Memorial Hospital Chicago Illinois USA; ^3^ Division of Cardiology, Bluhm Cardiovascular Institute, Department of Medicine Northwestern University Feinberg School of Medicine Chicago Illinois USA; ^4^ Department of Radiology Northwestern Memorial Hospital Chicago Illinois USA; ^5^ Ken & Ruth Davee Department of Neurology Northwestern University Feinberg School of Medicine Chicago Illinois USA

**Keywords:** cardiology, cardiothoracic surgery, infectious diseases, neurology, radiology & imaging

## Abstract

*Staphylococcus aureus* bacteremia (SAB) is particularly challenging when complicated by infective endocarditis (IE), metastatic spread, or evolving antimicrobial resistance. We describe a 76‐year‐old woman with poorly‐controlled diabetes who presented with bilateral knee septic arthritis and persistent methicillin‐sensitive *S. aureus* (MSSA) bacteremia complicated by in vivo emergence of oxacillin resistance despite a mecA‐negative genotype. Her clinical course was marked by an aortomitral intervalvular fibrosa abscess, vertebral osteomyelitis with epidural extension, a psoas abscess, and septic renal infarctions. She experienced breakthrough bacteremia despite initial clearance on oxacillin. Surgery was delayed due to intracranial hemorrhage but was ultimately enabled by middle meningeal artery embolization (MMAE). This case highlights the rare emergence of phenotypic oxacillin resistance in mecA‐negative MSSA during therapy and illustrates the role of MMAE in facilitating time‐sensitive cardiac surgery in the setting of recent intracranial bleeding. It underscores the importance of dynamic diagnostic reassessment, serial susceptibility testing, and multidisciplinary coordination in complicated SAB.

## Key Clinical Message

In *Staphylococcus aureus* bacteremia with high‐risk features, clinicians should pursue early transesophageal echocardiography, maintain vigilance for metastatic foci, and consider serial susceptibility testing to detect emerging beta‐lactam resistance—even in mecA‐negative isolates.

## 1 Introduction

*Staphylococcus aureus* remains a predominant cause of infective endocarditis (IE), with a well‐documented propensity for rapid hematogenous spread to distant sites such as joints, vertebral bodies, and the central nervous system [1, 2, 3]. Despite advances in antimicrobial therapy, *Staphylococcus aureus* bacteremia (SAB) continues to carry substantial morbidity and mortality, particularly when source control is delayed, metastatic foci develop, or host factors such as diabetes impair immune responses [4, 5, 6, 7, 8, 9]. Transthoracic echocardiography (TTE) lacks sensitivity for detecting vegetations, particularly periannular abscesses and posterior structures, making early transesophageal echocardiography (TEE) essential in high‐risk SAB [10, 11].

Dissemination further complicates management, especially in older adults and those with metabolic or immunologic comorbidities [12, 13, 14, 15]. Diagnostic delays frequently arise from nonspecific clinical presentations and overlap with other acute or chronic conditions; thus, a low threshold for MRI and other advanced imaging is critical for early identification of metastatic foci.

Therapeutic management may be further complicated by the rare but clinically significant emergence of antimicrobial resistance during treatment. Although methicillin‐sensitive *S. aureus* (MSSA) is typically beta‐lactam susceptible, non–mecA‐mediated resistance can rarely emerge in vivo, particularly in immunocompromised hosts [16, 17]. These factors highlight the importance of ongoing microbiological surveillance, including repeated blood cultures and susceptibility testing.

This case illustrates both classic features of IE alongside two distinctive elements: (1) in vivo emergence of oxacillin resistance in mecA‐negative MSSA, and (2) an uncommon application of middle meningeal artery embolization (MMAE) to stabilize intracranial hemorrhage and permit timely cardiac surgery. Together, these features highlight the importance of dynamic diagnostic reassessment and multidisciplinary coordination in complicated SAB.

## 2 Case History/Examination

A 76‐year‐old woman with poorly controlled type 2 diabetes mellitus (HbA1c 10.4%), hypertension, and recent transient ischemic attack presented to an outside hospital after a mechanical fall accompanied by a brief loss of consciousness. On arrival, she was febrile (102.9°F) and tachycardic (122 bpm) with elevated blood pressure (156/98 mmHg). Head CT revealed an acute subarachnoid hemorrhage (SAH) in the right frontal and parietal sulci, prompting transfer for neurosurgical evaluation.

Upon transfer, she was afebrile (98.5°F) but remained tachycardic (104 bpm) and hypertensive (164/78 mmHg). She endorsed bilateral knee pain and back pain. Physical examination revealed crusted, healing lesions on the right shoulder and upper back consistent with recent herpes zoster.

## 3 Differential Diagnosis, Investigations, and Treatments

Initial considerations for fever, bilateral knee pain, and back pain included infectious etiologies—such as septic arthritis, vertebral osteomyelitis/discitis with possible epidural abscess, and infective endocarditis—as well as inflammatory and noninflammatory musculoskeletal causes of articular pain. Traumatic musculoskeletal injury, given her recent fall, also warranted consideration early in the evaluation.

Laboratory studies demonstrated normocytic anemia (hemoglobin 8.6 g/dL), neutrophil‐predominant leukocytosis (WBC 9.6 × 10^9^/L; neutrophils 88.5%), markedly elevated inflammatory markers (ESR 92 mm/h; CRP 126.6 mg/L), and severe hyperglycemia (glucose 293 mg/dL). Urinalysis demonstrated glucosuria, proteinuria, and ketonuria, while renal function and coagulation parameters were normal.

Blood cultures on hospital day (HD) 2 grew methicillin‐sensitive *Staphylococcus aureus* (MSSA). Empiric vancomycin was initiated and later narrowed to cefazolin once susceptibilities returned. On the same day, the patient sustained a witnessed in‐hospital fall, with subsequent CT revealing a new 5‐mm subdural hematoma (SDH). Neurological examinations remained stable, and neurosurgery recommended conservative management with levetiracetam for seizure prophylaxis.

Persistent knee pain prompted bilateral arthrocentesis on HD 3. Synovial fluid analysis from both knees revealed elevated white blood cell counts (right: 17,000 cells/mm^3^; left: 31,000 cells/mm^3^, both > 90% PMNs), calcium pyrophosphate crystals, and MSSA growth—confirming septic arthritis with concomitant pseudogout. Spinal MRI, obtained for back pain, showed L3–L4 marrow edema and disc signal changes concerning for early discitis‐osteomyelitis. Knee irrigation and debridement were performed. Blood cultures cleared by HD 4.

**FIGURE 1:**
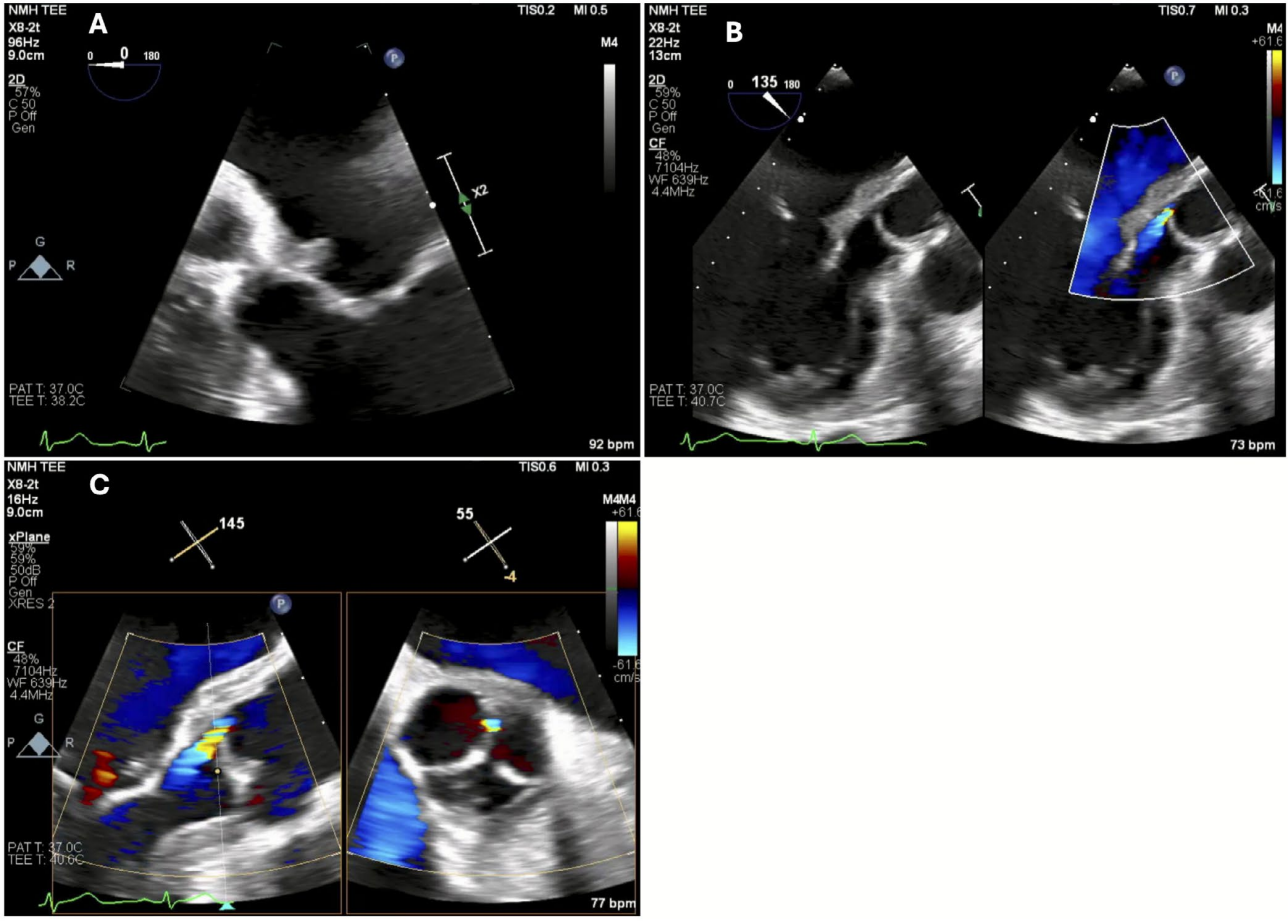
Two‐dimensional (B‐mode) transesophageal echocardiographic (TEE) still‐frames demonstrating: (A) a mobile echodensity (1.1 × 0.7 cm) attached to the anterior mitral annulus at the aortomitral curtain, consistent with vegetation; (B) heterogenous tissue extending into the aortic root, compatible with periannular abscess formation; and (C) mild aortic regurgitation originating between the left and non‐coronary cusps, with possible cusp perforation and a small filamentous structure on the aortic side of the valve suggestive of Lambl's excrescence or vegetation.

A transthoracic echocardiogram (TTE) obtained on HD 4 was nondiagnostic. Due to high‐risk features—diabetes, initial fever, and metastatic infection—a transesophageal echocardiogram (TEE) was pursued, confirming IE with periannular abscess (Figure 1A–C, Videos 1, 2, 3). Cefazolin was escalated to oxacillin for deep‐seated endovascular infection.

**VIDEO 1:** Corresponding transesophageal echocardiographic (TEE) cine clips demonstrating the dynamic features seen in Figure 1. Mobile echodensity (1.1 × 0.7 cm) attached to the anterior mitral annulus at the aortomitral curtain, consistent with vegetation. Video content can be viewed at https://onlinelibrary.wiley.com/doi/10.1002/ccr3.71888.

**VIDEO 2:** Heterogeneous echogenic material extending into the aortic root, compatible with periannular abscess formation. Video content can be viewed at https://onlinelibrary.wiley.com/doi/10.1002/ccr3.71888.

**VIDEO 3:** Color Doppler evidence of mild aortic regurgitation originating between the left and non‐coronary cusps, with suspected cusp perforation and a small filamentous structure on the aortic side of the valve, suggestive of Lambl's excrescence or vegetation. Video content can be viewed at https://onlinelibrary.wiley.com/doi/10.1002/ccr3.71888.

**FIGURE 2:**
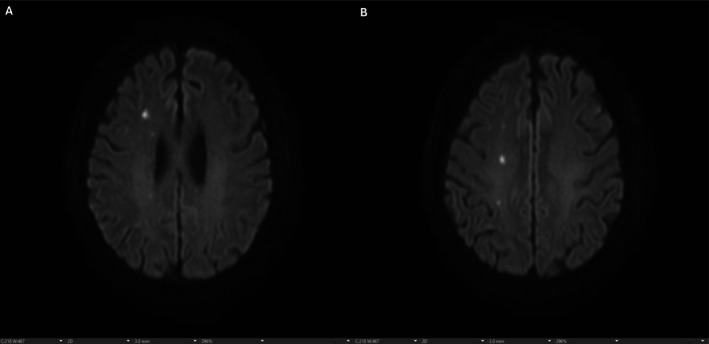
(A and B) Axial diffusion‐weighted MRI of the brain demonstrating multiple hyperintense foci within the cerebral parenchyma, consistent with septic emboli.

Further imaging revealed multiple acute and subacute cerebral infarcts on brain MRI (Figure 2), and CT of the abdomen/pelvis showed bilateral renal infarcts.

Cardiothoracic surgery was consulted for aortic root debridement and valve replacement, but recent intracranial hemorrhage precluded immediate intervention. To mitigate hemorrhagic risk and expedite surgical timing, bilateral middle meningeal artery embolization (MMAE) was performed on HD 15.

**FIGURE 3:**
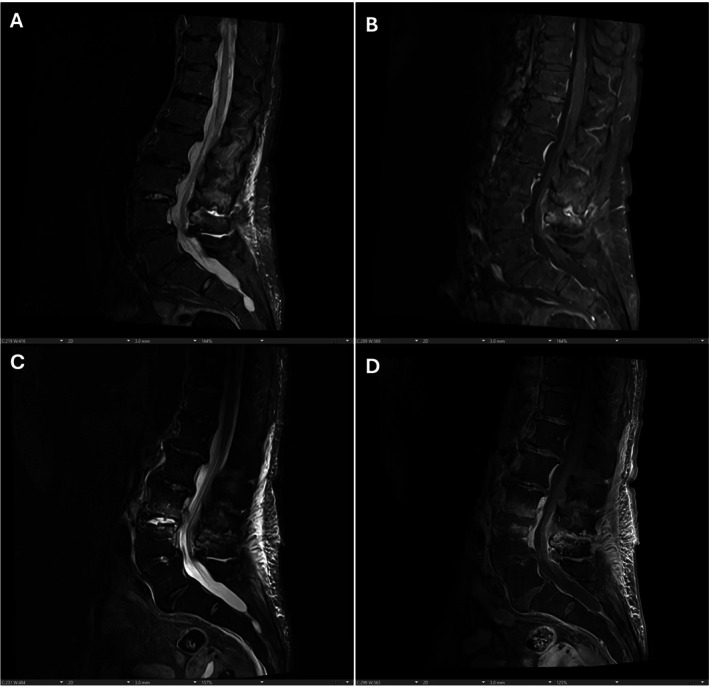
Sagittal MRI images of the lumbar spine comparing initial imaging (A and B) and repeat imaging approximately 1 month later (C and D), highlighting interval progression with increased T2‐weighted hyperintensity, greater enhancement, and worsening adjacent vertebral endplate edema at L3–L4, consistent with evolving discitis/osteomyelitis.

Despite targeted therapy, brain MRI later revealed numerous new infarcts in the right cerebral hemisphere and cerebellum. Lumbar MRI confirmed worsening L3–L4 discitis‐osteomyelitis (Figure 3), epidural phlegmon, right facet septic arthritis, and a 10‐mm right psoas abscess. Post‐MMAE imaging was stable, and the patient was cleared for surgery.

On HD 26, breakthrough bacteremia occurred, with blood cultures again growing *S. aureus* despite more than 3 weeks of oxacillin therapy. Vancomycin was restarted pending susceptibilities, which revealed new phenotypic oxacillin resistance (MIC ≥ 4 μg/mL) with negative mecA testing. Therapy was subsequently escalated to daptomycin for definitive treatment.

## 4 Outcome

A BioBentall procedure with extensive debridement of the aortomitral curtain abscess was performed. Intraoperatively, the patient developed ventricular tachycardia/fibrillation arrest requiring emergency resternotomy and internal defibrillation. No surgical bleeding or structural abnormalities were identified.

Postoperatively, she required inotropic support for transient right ventricular dysfunction. Echocardiography confirmed appropriate prosthetic valve function with only mild paravalvular regurgitation, and right ventricular function gradually improved. During recovery, she developed dysarthria, although neuroimaging revealed no new hemorrhage or infarct.

She continued on intravenous daptomycin for a planned 6–8‐week course. Supportive care included ventilatory support, diuresis for volume overload, glycemic control, and nutritional supplementation. At the time of this report, she remained critically ill but gradually improving, with no recurrent arrhythmias, reduced inotropic requirements, and participation in early physical rehabilitation. Major clinical events and antimicrobial decisions are summarized in Tables 1 and 2.

**TABLE 1:** Chronological clinical course summarizing key diagnostic, therapeutic, and neurosurgical events from admission through postoperative recovery.

HD	Event	Key findings
**Presentation & Initial Evaluation (HD 1–2)**		
1	Initial presentation after fall	Acute SAH; poorly controlled T2DM; healed zoster lesions (suspected portal of entry).
2	In‐hospital fall; bacteremia identified	New SDH. Blood cultures positive for MSSA. Vancomycin started.
**Septic Arthritis & Endocarditis Diagnosis (HD 3–6)**		
3	Arthrocentesis; spine MRI	Bilateral knee aspiration. Early L3–L4 signal changes. Cefazolin started.
4	Synovial fluid results; Knee I&D	CPPD crystals + MSSA in both knees. TTE nondiagnostic. Blood cultures clear.
5	Repeat TTE	TTE remains nondiagnostic; synovial/wound cultures confirm MSSA.
6	TEE → definitive endocarditis diagnosis	Vegetation at aortomitral curtain; intervalvular fibrosa abscess; mild AR with possible cusp perforation. CT Surgery consulted.
**Septic Emboli & Pre‐Operative Management (HD 7–15)**		
7	MRI brain	Septic emboli; new SDH/hygroma.
8	Antibiotic change; renal emboli	Cefazolin → oxacillin. Renal septic emboli identified.
8–14	Neurosurgical clearance period	Serial CT heads stable. Required 2 weeks before anticoagulation.
15	Bilateral MMAE	Performed to allow cardiac surgery safely.
**Disease Progression & Emergence of Resistance (HD 23–32)**		
23	Repeat MRI brain/spine	New septic infarcts; progression to L3–L4 discitis‐osteomyelitis; epidural phlegmon; R psoas abscess.
26	Breakthrough bacteremia	Persistent MSSA despite > 3 weeks oxacillin.
30	Resistance identified	Oxacillin MIC ≥ 4, mecA‐negative → β‐lactamase hyperproduction. Vancomycin started.
32	Switch to daptomycin	Persistent bacteremia → daptomycin, with clearance by HD 34.
**Cardiac Surgery & Post‐Operative Course (HD 37+)**		
37	BioBentall procedure	Aortic root replacement; abscess debridement; intraoperative VF arrest; resternotomy and defibrillation with ROSC.
38+	ICU recovery	Ventilation, vasopressors, improving hemodynamics. Daptomycin continued (planned 6‐week course).

*Note:* Hospital days (HD) reflect the progression from initial presentation with intracranial hemorrhage and MSSA bacteremia to multisystem dissemination, emergence of oxacillin resistance, and eventual aortic root surgery.

Abbreviations: CPPD, Calcium Pyrophosphate Dihydrate; HD, Hospital Day; I&D, Irrigation and Debridement; MMAE, Middle Meningeal Artery Embolization; MSSA, Methicillin‐Susceptible *Staphylococcus aureus*; SAH, Subarachnoid Hemorrhage; SDH, Subdural Hematoma; TEE, Transesophageal Echocardiogram; TTE, Transthoracic Echocardiogram.

**TABLE 2:** Sequence of antimicrobial therapy and corresponding clinical rationale.

Therapy phase	Antimicrobial regimen	Indication/rationale
Empiric therapy	Vancomycin (IV)	Initiated for suspected *S. aureus* bacteremia pending culture and susceptibility results.
Initial targeted therapy	Cefazolin (IV)	Narrowed after susceptibilities confirmed MSSA.
Optimized therapy	Oxacillin (IV)	Transitioned to an antistaphylococcal β‐lactam for optimal MSSA coverage; persistent bacteremia prompted further evaluation.
Empiric re‐escalation	Vancomycin (IV, brief reintroduction)	Reintroduced empirically after breakthrough bacteremia raised concern for resistance; discontinued once mecA‐negative oxacillin resistance confirmed.
Definitive therapy	Daptomycin (IV)	Instituted for oxacillin‐resistant, mecA‐negative *S. aureus* (β‐lactamase hyperproduction).

*Note:* The timeline reflects the progression from empiric to targeted treatment, emergence of β‐lactam resistance (mecA‐negative, β‐lactamase hyperproduction), and definitive management with daptomycin.

## 5 Discussion

This case exemplifies the diagnostic and therapeutic complexity of *Staphylococcus aureus* bacteremia (SAB), particularly when occurring in an elderly patient with significant metabolic and immunologic vulnerability. Multifocal infection, emerging resistance, and procedural constraints required repeated clinical reassessment and multidisciplinary coordination.

### 5.1 Diagnostic Challenges

A nondiagnostic TTE despite diabetes, fever, and metastatic infection highlights TTE's limitations in high‐risk SAB [10, 18, 19, 20]. This underscores a crucial diagnostic principle: in any case of sustained SAB, TEE should be employed early and unhesitatingly, even after apparent culture clearance, to evaluate for occult endocarditis or paravalvular complications.

The coexistence of pseudogout and septic arthritis risked diagnostic anchoring. However, this case reminds us that crystal‐induced inflammation does not preclude concurrent infection, particularly in patients with impaired immune function. Dual pathology is more than a curiosity; it is a diagnostic trap that delays source control. A high index of suspicion, guided by clinical context rather than cytologic purity, must prevail.

Furthermore, this case demonstrates how subtle or nonspecific symptoms—such as back pain or knee pain—should not be dismissed as peripheral or incidental concerns, as they may in fact herald deep‐seated infection, including vertebral osteomyelitis or epidural involvement. MRI remains the imaging modality of choice and should be employed liberally when musculoskeletal symptoms develop in the context of SAB—even when such symptoms follow recent trauma, which may predispose clinicians to diagnostic anchoring.

### 5.2 Unusual Resistance Evolution

Despite bilateral arthrotomy and appropriately targeted antibiotic therapy, the patient's bacteremia persisted and ultimately declared its resistance phenotype, shifting from oxacillin‐susceptible to oxacillin‐resistant *S. aureus* . This pattern reflects a growing recognition of non–mecA‐mediated oxacillin resistance, an emerging mechanism that may evade routine molecular detection. Such cases underscore the importance of repeated phenotypic susceptibility testing, including serial minimum inhibitory concentration (MIC) measurements, particularly when the patient's clinical course diverges from the expected response to therapy [21, 22, 23, 24].

### 5.3 Disseminated Complications

The multiplicity of metastatic complications reflects the aggressive endothelial tropism of *S. aureus* [25, 26, 27]. Early imaging in SAB with musculoskeletal or neurologic symptoms remains crucial to prevent delayed recognition of deep infection and to guide escalation of antimicrobial therapy and surgical planning.

### 5.4 Surgical Timing in the Setting of Intracranial Hemorrhage

The use of middle meningeal artery embolization (MMAE) to stabilize hemorrhagic risk and permit safe valve replacement is a testament to the evolving interface between neurology and cardiac surgery in managing septic complications. While MMAE is increasingly used for chronic subdural hematomas, its role in facilitating cardiac surgery in infective endocarditis is emerging, and this case contributes to growing anecdotal evidence supporting its use when conventional pathways are untenable.

### 5.5 Postoperative Course and Clinical Implications

The postoperative course—marked by right ventricular dysfunction, ventricular arrhythmias, respiratory decompensation, and neurologic uncertainty—reinforces a sobering clinical truth: even with optimal intervention, the trajectory of complicated SAB remains precarious. Yet, the patient's gradual improvement at the time of this report affirms the central role of persistence, multidisciplinary coordination, and individualized risk–benefit calculus.

## 6 Conclusions

SAB complicated by infective endocarditis, disseminated infection, and evolving antimicrobial resistance demands continual diagnostic reassessment and close multidisciplinary coordination. Even when initial imaging or early microbiologic responses appear reassuring, high‐risk features such as diabetes, persistent symptoms, or metastatic foci should prompt early TEE, comprehensive imaging, and sustained clinical vigilance. Incorporating serial susceptibility testing and molecular diagnostics can support earlier detection of resistance shifts and guide timely therapy modifications. As surgical timing in complicated IE remains challenging, emerging interventions (such as MMAE) may offer safe alternatives when standard algorithms fall short. Further research is needed to clarify the prevalence, triggers, and clinical impact of non‐mecA‐mediated resistance in MSSA, an underrecognized cause of treatment failure.

## Author Contributions

**Leandro Bosch:** conceptualization, investigation, project administration, visualization, writing – original draft, writing – review and editing. **Alexie Carletti:** conceptualization, resources, writing – review and editing. **Toluwalase Awoyemi:** conceptualization, data curation, validation, writing – review and editing. **Mohamed Al‐Kazaz:** resources, validation, visualization, writing – review and editing. **Alexander J. Nemeth:** resources, validation, visualization, writing – review and editing. **David Young:** supervision, validation, writing – review and editing.

## Funding

The authors have nothing to report.

## Ethics Statement

Per institutional policy at Northwestern University Feinberg School of Medicine, ethics committee approval is not required for single‐patient case reports that are de‐identified and presented with patient consent.

## Consent

Written informed consent for publication of the case and accompanying images was obtained from the patient.

## Conflicts of Interest

Dr. Mohamed Al‐Kazaz received a research grant from Kiniksa Pharmaceuticals, Ventyx BioSciences, and Cardiol Therapeutics, speaking honoraria from Kiniksa Pharmaceuticals, and consulting fees from Edwards Lifesciences. The other authors declare no conflicts of interest.

## Data Availability

All data necessary for this case report are available as part of this article and no additional source data are required.
